# Supplementary material for: The lived experiences of UK physiotherapists involved in Cauda Equina Syndrome litigation. A qualitative study
Source: PLoS One. 2023 Sep 14;18(9):e0290882. doi: 10.1371/journal.pone.0290882 (PMC10501620; doi:10.1371/journal.pone.0290882)
Supplement: S1 File — (DOCX) [file pone.0290882.s002.docx]

**Topic Guide**

Open questions that explored the following:

About you and your past role

- Clinican experience, who worked for, your role, experience in that role

About your experience of litigation

- Awareness of litigation
- Any litigation training – including what, when, stage in career, how much
- Personal experience of the litigation process – your story, feelings, support during litigation, impact of litigation
- Reflecting on own/ colleagues experience of litigation - what was done well and what could be improved

Anything else you to add
