# Supplementary material for: The lived experiences of UK physiotherapists involved in Cauda Equina Syndrome litigation. A qualitative study
Source: PLoS One. 2023 Sep 14;18(9):e0290882. doi: 10.1371/journal.pone.0290882 (PMC10501620; doi:10.1371/journal.pone.0290882)
Supplement: Attachment — Submitted filename: Response to Reviewers.docx [file pone.0290882.s003.docx]

**Editor’s comments**

Journal requirements:

Comment E1.

1. Please ensure that your manuscript meets PLOS ONE's style requirements, including those for file naming. The PLOS ONE style templates can be found at

[https://journals.plos.org/plosone/s/file?id=wjVg/PLOSOne_formatting_sample_main_body.pdf](https://protect-eu.mimecast.com/s/IFIrCWw6gilk9gXFnMqLT?domain=journals.plos.org) and

[https://journals.plos.org/plosone/s/file?id=ba62/PLOSOne_formatting_sample_title_authors_affiliations.pdf](https://protect-eu.mimecast.com/s/WNutCX7DjupJ5yDsm4AO0?domain=journals.plos.org)

Response E1

The manuscript has been checked to ensure it meets PLOS ONE's style requirements, including those for file naming.

Comment E2.

2. Please note that PLOS ONE has specific guidelines on code sharing for submissions in which author-generated code underpins the findings in the manuscript. In these cases, all author-generated code must be made available without restrictions upon publication of the work. Please review our guidelines at [https://journals.plos.org/plosone/s/materials-and-software-sharing#loc-sharing-code](https://protect-eu.mimecast.com/s/VeqtCYQEkcG81JpiZp3Jx?domain=journals.plos.org) and ensure that your code is shared in a way that follows best practice and facilitates reproducibility and reuse.

Response E2

Data derived codes were used in the analysis of the paper. A minimal data set has been provided to facilitate reproducibility and reuse. Author generated codes were not used as part of this research.

Comment E3.

3. Please provide additional details regarding participant consent. In the ethics statement in the Methods and online submission information, please ensure that you have specified (1) whether consent was informed and (2) what type you obtained (for instance, written or verbal, and if verbal, how it was documented and witnessed). If your study included minors, state whether you obtained consent from parents or guardians. If the need for consent was waived by the ethics committee, please include this information.

Response E3.1

The following text has been added to the manuscript (p6) and included in the online submission:

Informed consent was obtained verbally from all participants prior to participation. Participant consent was digitally audio-recorded by the interviewers.

If you are reporting a retrospective study of medical records or archived samples, please ensure that you have discussed whether all data were fully anonymized before you accessed them and/or whether the IRB or ethics committee waived the requirement for informed consent. If patients provided informed written consent to have data from their medical records used in research, please include this information.

Response E3.2

N/A

Comment E4

4. In your Data Availability statement, you have not specified where the minimal data set underlying the results described in your manuscript can be found. PLOS defines a study's minimal data set as the underlying data used to reach the conclusions drawn in the manuscript and any additional data required to replicate the reported study findings in their entirety. All PLOS journals require that the minimal data set be made fully available. For more information about our data policy, please see [http://journals.plos.org/plosone/s/data-availability](https://protect-eu.mimecast.com/s/eOsHCZw6liOG4V8CXtCjl?domain=journals.plos.org).

Upon re-submitting your revised manuscript, please upload your study’s minimal underlying data set as either Supporting Information files or to a stable, public repository and include the relevant URLs, DOIs, or accession numbers within your revised cover letter.

We will update your Data Availability statement to reflect the information you provide in your cover letter.

Response E4

We have uploaded the minimal data set underlying the results described in our manuscript to the university repository. This can be accessed via DOI [10.23634/MMU.00632413](https://protect-eu.mimecast.com/s/waXxC1YwmIvgkJRhLU-2S?domain=doi.org) (which will be made live on acceptance of the paper)

Comment E5.

5. Please review your reference list to ensure that it is complete and correct. If you have cited papers that have been retracted, please include the rationale for doing so in the manuscript text, or remove these references and replace them with relevant current references. Any changes to the reference list should be mentioned in the rebuttal letter that accompanies your revised manuscript. If you need to cite a retracted article, indicate the article’s retracted status in the References list and also include a citation and full reference for the retraction notice.

Response E5

The reference list has been reviewed to ensure it is complete and correct.


Comment E6

6. While revising your submission, please upload your figure files to the Preflight Analysis and Conversion Engine (PACE) digital diagnostic tool, [https://pacev2.apexcovantage.com/](https://protect-eu.mimecast.com/s/OxZVC6m8rcQDOA0HQqIvq?domain=pacev2.apexcovantage.com).

Response E6

Figure files (Fig 1) have been uploaded to PACE and have been included.

**Reviewers’ comments**

[Note: HTML markup is below. Please do not edit.]

Reviewers' comments:

Reviewer's Responses to Questions

**Comments to the Author**

1. Is the manuscript technically sound, and do the data support the conclusions?

The manuscript must describe a technically sound piece of scientific research with data that supports the conclusions. Experiments must have been conducted rigorously, with appropriate controls, replication, and sample sizes. The conclusions must be drawn appropriately based on the data presented.

Reviewer #1: Yes

Reviewer #2: Yes

Response – Thank you

2. Has the statistical analysis been performed appropriately and rigorously?

Reviewer #1: Yes

Reviewer #2: Yes

Response – Thank you

3. Have the authors made all data underlying the findings in their manuscript fully available?

The [PLOS Data policy](https://protect-eu.mimecast.com/s/s9OyC3j1ouLBAJ9spwWdj?domain=plosone.org) requires authors to make all data underlying the findings described in their manuscript fully available without restriction, with rare exception (please refer to the Data Availability Statement in the manuscript PDF file). The data should be provided as part of the manuscript or its supporting information, or deposited to a public repository. For example, in addition to summary statistics, the data points behind means, medians and variance measures should be available. If there are restrictions on publicly sharing data—e.g. participant privacy or use of data from a third party—those must be specified.

Reviewer #1: Yes

Reviewer #2: Yes

Response – Thank you

4. Is the manuscript presented in an intelligible fashion and written in standard English?

PLOS ONE does not copyedit accepted manuscripts, so the language in submitted articles must be clear, correct, and unambiguous. Any typographical or grammatical errors should be corrected at revision, so please note any specific errors here.

Reviewer #1: Yes

Reviewer #2: Yes

Response – Thank you

**Reviewer 1**

Comment R1_5.

5. Review Comments to the Author

Reviewer #1: Dear Authors,

This study addresses an important topic. I enjoyed reading this manuscript. This is an interesting and novel study about the litigation effect on several aspects of physiotherapist’s life and work. The authors described a robust, well-designed study and have reported some interesting results. That said, the manuscript in its present form has some shortcomings regarding the method that I have outlined below. I have also provided one specific comment for consideration.
Response – Thank you

Comment R1_5.1

Specific comment:
⎫ Abstract- method: Please add the COREQ as a criterion for qualitative research.
Response R1_5.1

This has been added (p2)

Comment R1_5.2

Methods

How did the themes and sub-themes refine by the research team? Please more explain about the process of identifying, selecting, and refining 4 main themes and several sub-themes.

Response R1_5.2

Additional text has been added to the manuscript (p7) as follows to provide further detail re the process:

This involved the team (XX, XX, XX) independently listening to the audio-recordings and reading the transcripts. Open coding was used to code the data. This involved reading each transcript line by line to identify salient text related to the research question. Data derived codes were used to summarise the data. Codes were recorded using Nvivo software (version 20.6.1). Patterns across the dataset were then iteratively explored, and theoretically cognate codes were grouped to create sub-themes. Conceptually similar sub-themes were grouped together into emergent themes independently by the research team. The themes were then discussed, critically reviewed, and refined by the research team (XX, XX, XX, XX, XX) to create the final themes. There was concordance in the themes identified by the team and any refinement of themes related to semantics. Preliminary analysis was undertaken after each interview, which iteratively fed into subsequent data generation.

Comment R1_5.3
Why didn’t you consider the litigation effects on married life, sexual life, and specially for women, the mother-child relationship? It seems they could be important and should be considered as sub-themes for litigation effect.

Response R1_5.3

Thank you. Gadamerian hermeneutic phenomenology was used to explore the participants’ lived experience. Using this approach, broad open questions were asked, and specific a priori questions, which may have biased the data generated, were avoided. The literature and PPI group informed the topics and the broad open questions used in the interviews. This included a broad open question about the personal impact on the participant’s wellbeing, with follow-up questions being asked in response to what the participant said, to explore their experience in greater depth. As participants in our study did not discuss or raise their married life, sexual life, and the mother-child relationship as being impacted by litigation, we could not include this as a sub-theme in this study.

**Reviewer 2**

Reviewer #2: 
Comment R2_6

Dear Authors

The manuscript was well-written. Thanks for submitting your manuscript. I have checked the qualitative checklist for your manuscript. All the requirements were provided. Please make sure to avoid dual publication.

Response R2_6

Thank you
